# From contact coverage to effective coverage of community care for patients with severe mental disorders: A real-world investigation from Italy

**DOI:** 10.3389/fpsyt.2022.1014193

**Published:** 2022-11-29

**Authors:** Giovanni Corrao, Matteo Monzio Compagnoni, Angelo Barbato, Barbara D’Avanzo, Teresa Di Fiandra, Lucia Ferrara, Andrea Gaddini, Alessio Saponaro, Salvatore Scondotto, Valeria D. Tozzi, Flavia Carle, Simona Carbone, Daniel H. Chisholm, Antonio Lora

**Affiliations:** ^1^National Center for Healthcare Research and Pharmacoepidemiology, University of Milano-Bicocca, Milan, Italy; ^2^Unit of Biostatistics, Epidemiology, and Public Health, Department of Statistics and Quantitative Methods, University of Milano-Bicocca, Milan, Italy; ^3^Department of Health Policy, Istituto di Ricerche Farmacologiche Mario Negri IRCCS, Milan, Italy; ^4^Previously General Directorate for Health Prevention, Italian Health Ministry, Rome, Italy; ^5^Center of Research on Health and Social Care Management, SDA Bocconi School of Management (Bocconi University), Milan, Italy; ^6^Agency for Public Health, Rome, Italy; ^7^General Directorate of Health and Social Policies, Bologna, Italy; ^8^Department of Health Services and Epidemiological Observatory, Regional Health Authority, Palermo, Italy; ^9^Center of Epidemiology and Biostatistics, Polytechnic University of Marche, Ancona, Italy; ^10^Department of Health Planning, Italian Health Ministry, Rome, Italy; ^11^Department of Mental Health and Substance Abuse, World Health Organization, Geneva, Switzerland; ^12^Department of Mental Health and Addiction Services, ASST Lecco, Lecco, Italy

**Keywords:** effective coverage, mental healthcare, health service research, quality of healthcare, healthcare utilization database

## Abstract

**Objectives:**

To measure the gap between contact and effective coverage of mental healthcare (MHC).

**Materials and methods:**

45,761 newly referred cases of depression, schizophrenia, bipolar disorder, and personality disorder from four Italian regions were included. A variant of the self-controlled case series method was adopted to estimate the incidence rate ratio (IRR) for the relationship between exposure (i.e., use of different types of MHC such as pharmacotherapy, generic contact with the outpatient services, psychosocial intervention, and psychotherapy) and relapse (emergency hospital admissions for mental illness).

**Results:**

11,500 relapses occurred. Relapse risk was reduced during periods covered by (i) psychotherapy for patients with depression (IRR 0.67; 95% CI: 0.49 to 0.91) and bipolar disorder (0.64; 0.29 to 0.99); (ii) psychosocial interventions for those with depression (0.74; 0.56 to 0.98), schizophrenia (0.83; 0.68 to 0.99), and bipolar disorder (0.55; 0.36 to 0.84), (iii) pharmacotherapy for patients with schizophrenia (0.58; 0.49 to 0.69), and bipolar disorder (0.59; 0.44 to 0.78). Coverage with generic care, in absence of psychosocial/psychotherapeutic interventions, did not affect risk of relapse.

**Conclusion:**

This study ascertained the gap between contact and effective coverage of MHC and showed that administrative data can usefully contribute to assess the effectiveness of a mental health system.

## Introduction

Provision of appropriate care through delivery of quality health services to people in need is a core function of health systems. Accurate measurement of health service outputs and related outcomes is essential for tracking performance and addressing decision processes. One measure to determine how a program is performing is the coverage level it achieves (1). However, it has been found that increasing use of health services do not always translate into health gains (2). Indeed, although coverage is important, it does not always reflect the quality of the care provided, the extent to which key treatments are implemented as planned and consequently the actual health outcomes resulting from adequate treatments (3). Distinct from the measurement of contact coverage with which provision of care is usually monitored, it is therefore useful to identify the rate of effective coverage (4).

The World Health Organization includes the provision of mental healthcare in community-based settings as a key objective of Mental Health Care (MHC) systems (5). As a country, Italy offers a unique opportunity to explore community-oriented MHC. Indeed, since the approval of the psychiatric reform law in 1978 (6), a process of deinstitutionalization led to a profound shift from hospital-based models of care to community-oriented mental health services (7).

The conceptualization developed by Tanahashi et al., in their pivotal framework (8), can be very useful for addressing what and how monitoring and assessing coverage of community-oriented mental health services. In particular, the concepts of “potential coverage” (i.e., services availability, accessibility, and acceptability), “contact coverage” (i.e., the gap between use and need), and “effective coverage” (i.e., the gap between the use of service and the health gain) should drive the development of indicators for measuring the system functioning. Furthermore, in 2003, the World Health Organization resumed and re-introduced the concept of effective coverage, and its measurement was suggested to be incorporated into health system performance assessment (9).

Thus, to monitor the extent to which mental health system meets key objectives, the Italian Ministry of Health developed a system of measurement of MHC performance with the so-called QUADIM-MAP project. However, because better contact or treatment coverage does not necessarily mean more effective coverage (1, 4, 10), a study for evaluating the association between MHC coverage and measurable clinical outcomes was designed. In other terms, our study focused on measuring the gap between service use and health gain (i.e., the “effective coverage” according with the above reported conceptualization), with particular focus on identifying which services do not offer any evidence of generating health benefits to patients who used they. We also focused on components of service use and health outcomes measurable with available administrative data. This is at the same time the challenge of our project (being its aim to build indicators able to capture the components of service use with effective health implications) but also its limitation (being the measurements restricted to available data, and building surrogate measures was therefore necessary). The current paper reports methods and main findings regarding a large sample of Italian patients newly taken-into-care by the Departments of Mental Health (DMHs) of the National Health System (NHS) with diagnosis of depressive, schizophrenic, bipolar and personality disorder [i.e., the mental disorders with greatest clinical relevance and the highest burden for national health services (11–14)].

## Materials and methods

### Data sources

The QUADIM-MAP project is based on computerized Healthcare Utilization (HCU) databases from the Italian regions of Lombardy (northwest), Emilia-Romagna (northeast) and Lazio (central), and the province of Palermo (southern Italy) (15). Overall, data covered nearly 37% of the entire Italian population.

In Italy, all citizens have equal access to healthcare provided by the National Health Service (NHS). An automated system of HCU databases is used to locally manage health services in each region (16). HCU data include a variety of information on residents, such as diagnosis at discharge from public or private hospitals, outpatient drug prescriptions, specialist visits and diagnostic exams provided fully or partly free-of-charge by the NHS. In addition, a specific automated system concerning mental health care gathers data from regional Departments of Mental Health (DMHs) accredited by the NHS. The system provides demographic information, and diagnostic and therapeutic codes for patients receiving specialist MHC. These various types of data can be interconnected, since a unique individual identification code is used in all databases for each NHS beneficiary. Due to privacy issues, each identification code is automatically anonymized and the inverse process is only allowed to the Regional Authority upon request of judicial Authorities. Further details on HCU database in the field of MHC have been reported elsewhere (15, 17–19). Diagnostic and drug therapy codes used for drawing records and fields from the considered databases are reported in the Supplementary Table 1.

### Cohort selection and follow-up

The target population consisted of all NHS beneficiaries resident in Lombardy, Emilia-Romagna, Lazio, and Palermo, aged 18–65 years (13.5 million inhabitants) (15, 19). NHS beneficiaries resident in these areas, who during the recruitment period had at least one contact with a mental health service (MHS) and had a diagnosis of depression, schizophrenia, bipolar disorder, or personality disorder, were identified, and labeled as prevalent cases. According to data availability, distinct recruitment periods were considered, specifically: from January 2013 to December 2016 for Lombardy, from January 2015 to December 2016 for Emilia-Romagna and Palermo, and from January 2015 to December 2015 for Lazio.

With the aim of including individuals newly taken-into-care by the NHS who had for the first time a diagnosis of the considered disorders, prevalent cases who received a diagnosis of mental disorder at any time prior the recruitment period, and those who experienced at least one hospital admission to a psychiatric ward or received at least two prescriptions of drug therapies for their treatment in the years 2013–2014 (or 2011–2012, for Lombardy Region only), were excluded. In addition, under the assumption that schizophrenic, bipolar and personality disorder may hardly be diagnosed for the first time later than the age of 40 years (20), patients aged 41 years or older at the time of diagnosis were excluded. The same criterion was not adopted for depression, since in this case new diagnoses among individuals older than 41 years cannot be excluded. The remaining patients represented the study cohort and were labeled as patients newly taken-into-care for mental disorders. Cohort members accumulated person-time of follow-up from the date of diagnosis, until death, emigration or endpoint of follow-up whichever came first. According to data availability, different final dates were considered, specifically June 30, 2018 for Lombardy, Emilia-Romagna and Palermo, and December 31, 2016 for Lazio.

### Outcome assessment

Emergency admissions to psychiatric ward with diagnosis of mental disorder that occurred during follow-up were recorded for each cohort member. They were labeled as outcome episodes and were considered as measurable surrogates of relapse (21, 22).

### Measuring exposure to mental health care

Exposure to two broad categories of MHC was considered, specifically drug treatments and community care interventions. Drug treatments correspond to dispensation of drugs by the MHS or retail pharmacies and included antidepressants for depression, antipsychotics for schizophrenia, and mood stabilizers (lithium, lamotrigine, valproic acid, carbamazepine, and second-generation antipsychotics) for bipolar disorder and all the three classes of drugs (i.e., antidepressants, antipsychotics, and mood stabilizers) for personality disorder. Time coverage of each dispensed drug treatment was established according to the defined-daily-dose (DDD) metric.^1^

Community care interventions included whichever contact with the Community Mental Health Centers, Psychiatric outpatient Clinics and Day Care Centers resulting in at least one of the twenty-one interventions coded in the Italian Mental Health Information System and listed in Supplementary Table 2. Of the twenty-one interventions we identified those which we appointed as (i) Psychosocial non-psychotherapeutic interventions (Individual and group living skills training, Individual and group socialization, Single family and multifamily psychoeducation, Individual and group bodywork, Work training, and Assistance with financial and welfare procedures) and (ii) Psychotherapy sessions (Psychological interview, and individual, couple, family, and group psychotherapy). By exclusion, the remaining interventions were considered as Generic care (Psychiatric visit, Meeting with a professional, Support, Meeting with relatives, Consultation, Medico-legal assessment). The time coverage was established by assuming at least one intervention every 30 days.

In this way, the entire pathway of MHC experienced by each cohort member was built. Starting from the first month following the date of diagnosis, we calculated the monthly rate of coverage with drug treatment and community care interventions during the entire follow-up. As far as community care is concerned, we considered separately the rate of coverage with interventions classified under the heading of generic care, psychosocial non-psychotherapeutic interventions, and psychotherapy sessions, their sum being denoted as monthly rate of overall community care coverage.

### Additional measurements

Baseline characteristics of cohort members comprehended gender, age, years of education, employment status, marital status, and living arrangements. Furthermore, patients were categorized according to the Multisource Comorbidity Score (MCS), a new index of patients’ clinical status derived from (i) inpatients diagnostic information and (ii) outpatient drug prescriptions, provided by the regional Italian data and validated for outcome prediction (23, 24).

### Self-controlled case-referent series design

To control for patient specific characteristics while investigating the risk of relapse, we used a self-controlled case series (SCCS) design (25–30). This method uses a within person approach to compare the rates of relapse while an individual patient was covered or uncovered by MHC. With the aim of accounting for assumption violations, the following devices were adopted. First, a 90-day time-window after each relapse occurrence was excluded for taking into account the possibility that relapse onset affects the subsequent exposure process. Second, a 180-day time-window before each relapse occurrence was also excluded to take into account the so-called protopathic bias (31, 32). Shortly, as an increased MHC is expected whenever the patient’s symptomatology worsens (i.e., the true but unmeasurable outcome of interest), and because the latter is expected to increase the risk of emergency admissions to psychiatric ward (i.e., the measured outcome surrogating the true one), the increased use of mental health services could be wrongly interpreted as the paradoxical effect of the care in increasing the risk of relapse. For this reason, a lag-time period of 180 days immediately preceding the detected outcome was applied to ignore coverage with mental health services which may generate protopathic bias (31).

Third, a self-controlled referent series was built to consider the possibility that exposure and relapse is time-correlated (33). In other terms, as a concentration of both MHC and relapses is expected early after diagnosis, an artificial positive exposure to MHC → relapse association should be observed for the easy reason that exposure and relapse are time-correlated. We tried to control for this source of bias by means of an original approach consisting in comparing estimates generated from the self-controlled case series, with those generated from a self-controlled referent series. This was made by randomly selecting from the same cohort that generated the case series, cohort members who during the observational time-window experienced both periods covered and periods uncovered by exposure to MHC, but did not experience any relapse episode. Case and referent series were individually matched for gender, age at cohort entry, and date of mental health disorder diagnosis. Therefore, the current paper used a modified version of the original SCCS design, which we called self-controlled case-referent series (SCCRS) design. Footnote of Supplementary Figure 1 provides further details of the adopted design.

Conditional Poisson regression was used for estimating incidence rate ratios, and corresponding 95% confidence intervals (CI), for both case (IRR_c_) and referent (IRR_r_) series. Because referent patients did not experience relapse, IRR_r_ estimates the portion of IRR_c_ due to change in therapeutic strategy. By dividing IRR_c_ by IRR_r_, the time-trend adjusted IRR_a_ was obtained. Time-varying covariates were considered in the models by including coverage rates of MHC and no MHC services (e.g., hospital admissions, drug prescriptions and outpatient services).

The SAS Software (version 9.4; SAS Institute, Cary, NC, USA) was used to perform the analyses. For all hypotheses tested, two-tailed *p*-values less than 0.05 were considered significant.

### Sensitivity analyses

Owing to the arbitrariness of time-window widths anticipating and following relapse, other widths were investigated in secondary analyses. Furthermore, robustness of the principal findings were investigated by only considering the first relapse episode (i.e., recurrent relapses were excluded from the analyses), as a recommended approach by Petersen et al., when recurrent events cannot be assumed independent (27). Finally, the separate effect of antidepressants, antipsychotics, and mood stabilizers on the risk of relapse in patients with diagnosis of personality disorders was investigated.

## Results

### Patients

**FIGURE 1:**
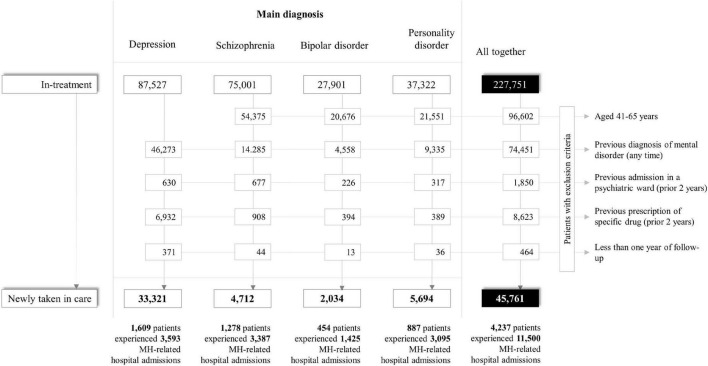
Flow-chart of inclusion and exclusion criteria for patients with severe mental health disorders. QUADIM-MAP project, Italy, 2013–2018.

The process of cohort selection is shown in Figure 1. Among the 227,751 eligible prevalent cases, 181,990 were excluded (mostly because of a previous diagnosis of mental disorder), while 45,761 individuals met the inclusion criteria and were included into the study cohort as newly taken-into-care patients with diagnosis of depression (73%), personality disorder (12%), schizophrenia (10%), or bipolar disorder (4%). Overall, 4,237 cohort members experienced the relapse at least once during follow-up, with a total number of 11,500 relapse episodes. The rate of relapse was higher among patients with schizophrenia and bipolar disorder (225 and 214 episodes every 1,000 person-months), slightly lower among patients with personality disorder (167 episodes every 1,000 person-months), and clearly lower among patients with depression (35 episodes every 1,000 person-months).

The baseline characteristics of newly taken-into-care cohort members are shown in Table 1. The mean age (SD) of patients with depression, schizophrenia or bipolar disorder and personality disorder was 46.5 years (11.9), 30.0 (6.5), 31.0 (6.5), and 28.9 (7.1) years, respectively. As expected, patients with depression were predominantly women, while the opposite occurred for patients with schizophrenia. Patients with depression and bipolar disorder had higher education and a worse clinical profile.

**TABLE 1:** Baseline characteristics of patients newly taken-into-care for severe mental disorders according with the main diagnosis. QUADIM-MAP project, Italy, 2013–2018.

	Depression (*N* = 33,321)	Schizophrenia (*N* = 4,712)	Bipolar disorder (*N* = 2,034)	Personality disorder (*N* = 5,694)
**Gender**				
Men	12,724 (38.2%)	3,108 (66.0%)	1,021 (50.2%)	2,831 (49.7%)
Women	20,597 (61.8%)	1,604 (34.0%)	1,013 (49.8%)	2,863 (50.3%)
**Age (years)**				
Mean (SD)	46.5 (11.9)	30.0 (6.5)	31.0 (6.5)	28.9 (7.1)
18–30	4,488 (13.5%)	2,542 (54.0%)	909 (44.7%)	3,309 (58.1%)
31–40	6,382 (19.2%)	2,170 (46.1%)	1,125 (55.3%)	2,385 (41.9%)
41–49	8,396 (25.2%)	–	–	–
51–64	14,055 (42.1%)	–	–	–
**Education years**				
0–5	6,235 (18.7%)	908 (19.3%)	306 (15.0%)	937 (16.4%)
6–8	11,141 (33.4%)	1,812 (38.5%)	701 (34.5%)	2,136 (37.5%)
9–13	9,453 (28.3%)	1,242 (26.4%)	646 (31.8%)	1,703 (29.9%)
≥ 14	3,002 (9.0%)	297 (6.3%)	201 (9.9%)	339 (6.0%)
Missing data	6,490 (19.6%)	453 (9.6%)	180 (8.9%)	579 (10.1%)
**Employment status**				
Employed	16,077 (48.2%)	1,513 (32.1%)	849 (41.7%)	2,062 (36.2%)
Unemployed	11,643 (34.9%)	2,526 (53.6%)	908 (44.6%)	2,840 (49.9%)
Retired (Invalid?)	2,051 (6.2%)	168 (3.6%)	74 (3.6%)	186 (3.3%)
Missing data	3,550 (10.7%)	505 (10.7%)	203 (10.0%)	606 (10.6%)
**Living arrangements**				
Family	3,855 (11.6%)	3,221 (78.1%)	1,367 (77.3%)	3,774 (76.0%)
Residential facility	16,004 (48.0%)	160 (3.9%)	64 (3.6%)	221 (4.4%)
Alone	3,466 (10.4%)	285 (6.9%)	170 (9.6%)	436 (8.8%)
Missing data	9,996 (30.0%)	459 (11.1%)	167 (9.5%)	538 (10.8%)
**Marital status**				
Married	15,203 (45.7%)	640 (13.6%)	420 (20.7%)	648 (11.4%)
Never married	10,129 (30.4%)	3,592 (76.2%)	1,382 (67.9%)	4,349 (76.4%)
Separated/Divorced	4,226 (12.7%)	117 (2.5%)	71 (3.5%)	237 (4.2%)
Widowed	930 (2.8%)	5 (0.1%)	7 (0.3%)	7 (0.0%)
Missing data	2,833 (8.4%)	358 (7.6%)	154 (7.6%)	453 (8.0%)
**Clinical profile†**				
Optimal	18,729 (56.2%)	2,531 (53.7%)	1,056 (51.9%)	3,716 (65.3%)
Good	9,759 (29.3%)	401 (8.5%)	287 (14.1%)	1,093 (19.2%)
Intermediate	3,085 (9.3%)	1,650 (35.0%)	590 (29.0%)	667 (11.7%)
Poor	1,748 (5.2%)	130 (2.8%)	101 (5.0%)	218 (3.8%)

^†^The clinical profile was assessed by the Multisource Comorbidity Score (MCS) according to the hospital admission and the drugs prescribed in the 2-year period before the index date.

Four categories of clinical status were considered: optimal (score = 0), good (1 ≤ score ≤ 5), intermediate (6 ≤ score ≤ 10), and poor (score ≥ 11).

### Mental health care

**FIGURE 2:**
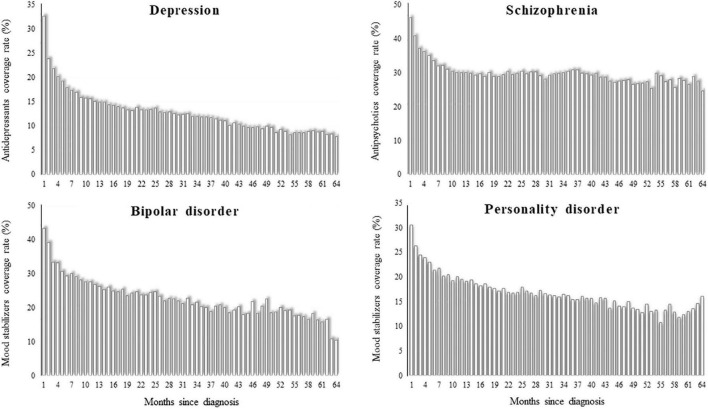
Monthly coverage rate of drug therapy from the first until the 64th month after diagnosis of depression, schizophrenia, bipolar disorder, and personality disorder. QUADIM-MAP project, Italy, 2013–2018.

Monthly rates of drug coverage were higher for patients with schizophrenia and bipolar disorder than for those with depression and personality disorder, being 46, 43, 33, and 31% of them, respectively, on drug therapy in the first month after diagnosis (Figure 2). There was, however, a gradual fall in the coverage rates across time, being patients in drug treatment after 5 years since diagnosis around 10% among those with depression and bipolar disorder, 15% among those with personality disorder, and 30% among those with schizophrenia.

**FIGURE 3:**
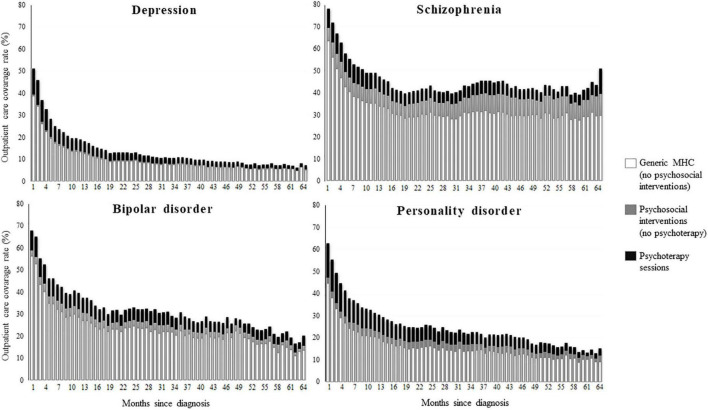
Monthly coverage rate of contacts with the mental health service from the first until the 64th month after diagnosis of depression, schizophrenia, bipolar disorder, and personality disorder. Coverage was assigned to psychotherapy whether at least one psychotherapeutic session was performed, to psychosocial intervention whether at least any other psychosocial intervention was performed, to generic mental health care whether the patient attended the service without receiving neither psychosocial nor psychotherapeutic intervention. QUADIM-MAP project, Italy, 2013–2018.

Monthly rates of community care coverage in the first month after diagnosis were 78% for patients with schizophrenia, 68% for those with bipolar disorder, 63% for those with personality disorder, and 51% for those with depression (Figure 3). Between 71 (personality disorder) and 83% (bipolar disorder) of these patients received generic MHC. The 93, 85, 77, and 59% of total psychosocial interventions consisted of psychotherapy sessions among patients respectively with depression, personality disorder, bipolar disorder, and schizophrenia. There was again a general fall in coverage rates, with only 10% of patients with depression, 15% with personality disorder, and 20% with bipolar disorder still in contact with services 5 years after diagnosis. Patients with schizophrenia had longer contacts, with a coverage rate around 50%.

### Mental health care and relapse

**FIGURE 4:**
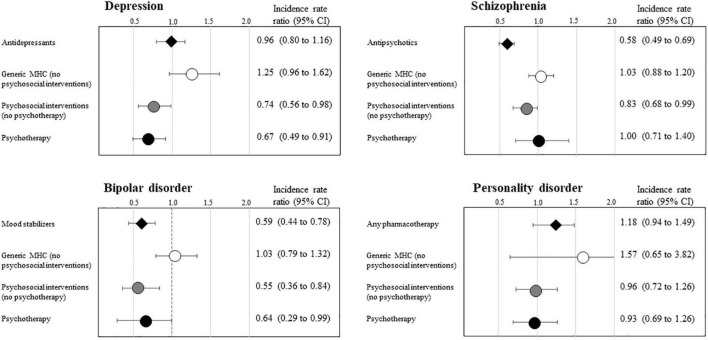
Self-controlled case-referent series estimates of the incidence rate ratio of relapse episodes associated with categories of mental care, according with the main diagnosis of depression, schizophrenia, bipolar disorder, and personality disorder. QUADIM-MAP project, Italy, 2013–2018. Self-controlled case-referent series incidence rate ratio, and 95% confidence interval, estimated with Poisson regression contrasting within-patient incidence of relapse onset observed during mental care coverage and no coverage person-time. Relapse was emergency hospital admissions in a psychiatric ward. Estimates were obtained through the design shown in Supplementary Figure 1; please see the extensive footnote of Supplementary Figure 1 for details about the design. Time-windows with width of 180 and 90 days respectively prior and following the relapse onset were removed for mitigating the effect of incorrect allocate person-time of MHC coverage.

Figure 4 shows that the relapse risk was reduced during periods covered by psychotherapy for patients with diagnosis of depression and bipolar disorder, by other psychosocial interventions for patients with diagnosis of depression, schizophrenia and bipolar disorder, by antipsychotics for patients with diagnosis of schizophrenia, and by mood stabilizers for patients with diagnosis of bipolar disorder. There was no evidence that the relapse risk was reduced by coverage with generic care, in the absence of psychosocial interventions, nor that patients with diagnosis of personality disorder may benefit by any care. Finally, there was no evidence that patients with diagnosis of depression and personality disorder did benefit from coverage with antidepressants or any psychotropic drugs, respectively.

### Sensitivity analyses

The lack of evidence that drug therapy prevents relapses in patients with personality disorder concerned not only the three drug classes considered together, but also each of them separately (Supplementary Table 3). The relationships described above did not substantially change by varying the time-window widths anticipating and following relapses, nor considering only the first relapse episode (Supplementary Table 4).

## Discussion

The main goal of this study was to assess to what extent effective coverage is achieved in a community based mental health system and to identify the factors associated with effective coverage. With concern to the association between pharmacotherapy and relapse prevention, we did not find that coverage with the investigated drugs was effective for preventing relapse in patients with depression. At first sight, this finding seems inconsistent with guidelines from Europe and the United States, typically recommending antidepressant medication use with or without psychotherapy for patients diagnosed with major depression (34). Although we cannot exclude that coverage with antidepressants might be effective for an unknown portion of patients with severe depression in our sample, we must however consider that, due to several flaws such publication and reporting bias, effectiveness of antidepressants for preventing relapse is systematically overestimated in the literature (35). In accordance with the extensive literature available (36–38), effectiveness of antipsychotics and mood stabilizers for relapse prevention in patients with schizophrenic and bipolar disorder was confirmed in our study. Finally, consistently with both the UK (NICE) and Australian guidelines, we confirmed that pharmacotherapy does not seem to be effective in preventing relapses in the course of personality disorders (39, 40).

Our study did not find that, in the absence of psychosocial/psychotherapeutic interventions, the use of mental health services might *per se* prevent the occurrence of relapse. This result is somewhat unexpected, as regular attending outpatient mental health services was supposed to surrogate for overall health-seeking behavior, therefore reducing the risk of relapse onset. Rather, we observed that relapse episodes occurred less frequently among patients with depression, schizophrenia, and bipolar disorder when they were offered psychosocial interventions, and among those with depression and bipolar disorder when they were offered psychotherapy. These findings are consistent with available evidence suggesting psychotherapy and psychosocial interventions, such as cognitive behavioral therapy and psychoeducation, for treatment of schizophrenia (15, 37, 41, 42) and a variety of psychotherapy models for depression (43–45). Currently, the data supporting the usefulness of specific psychotherapies for treatment of bipolar disorder are more limited (45, 46). Conversely, our observation that patients with personality disorder did not benefit of relapse reduction from coverage with either pharmacotherapy, psychosocial interventions psychotherapy, was in contrast with several guidelines recommending psychotherapy as primary, or core, treatment for this group of disorders (39, 40).

These findings should not be taken as robust evidence for the best community care for patients with severe mental illness. Beside the common pitfalls of observational studies, our study investigated the average effect of broadly defined interventions (irrespective of the type of drug, psychosocial intervention and psychotherapeutic approach employed) on patients considered as a homogeneous set of patients with severe mental disorders. Rather, our findings support the importance of monitoring the performance of community-based mental health services. In accordance with the landmark paper of Tanahashi (8), our study showed that the rate of generic use of care by MHS, is not sufficient for protecting patients from relapse risk. Conversely, effective coverage, i.e., ensuring coverage with pharmacotherapy and/or psychosocial interventions or psychotherapy targeted to specific disorders, showed evidence of prevention from relapse occurrence. Overall, our study shows that in Italy only a modest portion of the patients taken in care by community mental health services received effective care for relapse prevention.

Our study found that adherence to treatment was very poor among patients newly taken in care for severe mental disorders, in particular for those with depression. Coverage with antidepressant drug therapy and with at least one MHS contact respectively concerned 33 and 51% of patients 1 month after diagnosis of depression and fell to less than 10% for both the categories of MHC 5 years after diagnosis. This may be because several episodes of depression are short lasting or require brief treatments. Patients with schizophrenia maintained more contacts, although still scarce, since drug and MHC coverage respectively started from 46 to 78% 1 month after diagnosis and fell to 30 and 50% 5 years later. Intermediate values were observed for patients with bipolar and personality disorder. These findings were expected since very poor adherence to pharmacotherapy has been described for patients with depression (46, 47), schizophrenia (15, 48, 49), and bipolar disorders (50, 51). At our best knowledge, however, adherence to pharmacotherapy among patients with personality disorder had been never investigated, as well as there is a lack of direct comparisons of adherence between patients with different mental health disorders. Furthermore, studies mostly focused on adherence to pharmacotherapy, while other aspects of mental health service use once a patient was taken in care have been seldom considered (52–54).

The present study is unique in several respects. First, the investigation is based on data from a large, unselected population, which was made possible since in Italy a free healthcare system covers all citizens. In particular, the availability of high-quality individual data on outpatient and inpatient services supplied by the NHS, which, since about 10 years, can be linked to data on care provided by mental health departments (the so-called Italian Mental Health Information System), offers the opportunity to investigate large unselected populations, and to generate real-world evidence on mental health care (15). Second, our data reflect routine clinical practice, and are not affected by selective participation and recall bias (17). Third, patients were identified from the day of their first visit with the mental health service in which diagnosis of mental illness was made, and the complete pathway of mental healthcare services provided was known. Fourth, the SCCS design, and its modifications for accounting assumption violations, allowed to take into account both known and unknown time invariant confounders (including factors like drug abuse, or stigma to look for treatment). Potential time-varying confounders were also considered to limit residual confounding due to differences between periods covered and uncovered by mental health care. Finally, a number of sensitivity analyses confirmed the robustness of our findings.

Limitations of this study should be considered to correctly portray our results. Both exposure and relapse misclassification likely affected our estimates. Common sources of exposure misclassification include treatments dispensed by private services, as well as out-of-pocket payments. The psychiatric diagnosis itself could be misclassified (55). Outcome misclassification might be due to our inability to capture all relapse episodes, but only those requiring hospital admissions. In addition, residual time-varying confounding cannot be excluded in spite the great attention for avoiding it.

Finally, the main weakness of our study is that our conclusions only refer to MHC able of preventing detectable relapse episodes. This is an important limitation because, owing the narrowness of available data, we cannot speculate on the effect of care coverage on unmeasured outcome such as functional improvement following initial entry into care. There is therefore urgent need of integrating current informative systems with more complete data collection for better investigates all components of mental healthcare coverage.

In conclusion, our study found that the gap between contact and effective coverage in mental health is substantial. Community mental healthcare showing evidence of effectively prevent the onset of relapse were psychosocial interventions and psychotherapy for depression, antipsychotics and psychosocial intervention for schizophrenia, mood stabilizers, psychosocial intervention, and psychotherapy for bipolar disorder. Reasons of lack of evidence offered by our study that the onset of relapse episodes was affected by coverage neither with antidepressants for patients with depression, nor with any intervention in patients with personality disorder, should be urgently clarified. The current study supplied evidence that administrative data may contribute to assessing the effectiveness of a mental health system even in the absence of *ad hoc* data collection.

## Data availability statement

The datasets presented in this article are not readily available because the data that support the findings of this study are available from the Regions of Lombardy, Lazio and Emilia-Romagna, the Province of Palermo, but restrictions apply to the availability of these data, which were used under license for the current study, and so are not publicly available. Data are however available from the authors upon reasonable request and with permission of the Regions involved in this study. Requests to access the datasets should be directed to AG, andrea.gaddini@gmail.com; AS, Alessio.Saponaro@regione.emilia-romagna.it; SS, salvatore.scondotto@regione.sicilia.it; GC, giovanni.corrao@unimib.it; and Francesco Bortolan, francesco_bortolan@regione.lombardia.it.

## Ethics statement

The studies involving human participants were reviewed and approved by Ethical Committee of the University of Milano-Bicocca (Protocol number 497, Year 2019). Written informed consent for participation was not required for this study in accordance with the national legislation and the institutional requirements.

## “QUADIM project” working group

“QUAlità dei servizi per la cura dei DIsturbi Mentali gravi” QUADIM working group (Italian Health Ministry, Prevention Dept): Italian Ministry of Health, General Directorate for Health Prevention: Di Fiandra T, Magliocchetti N. Department of Mental Health, Lecco Hospital, Lecco, Italy: Lora A, Barri M. Emilia-Romagna Region: Saponaro A. Lazio Region: Gaddini A, Mattia V. Sicily Region: Scondotto S, Pollina Addario W, Berardi M, Di Giorgi M. University of Milano-Bicocca, Laboratory of Healthcare Research and Pharmacoepidemiology: Corrao G, Monzio Compagnoni M. IRCCS Mario Negri: Barbato A, D’Avanzo B, Monti I. SDA Cergas Bocconi: Tozzi V.D., Ferrara L.

## “Monitoring and assessing diagnostic-therapeutic paths (MAP) project” working group

“Monitoring and assessing diagnostic-therapeutic paths” MAP working group (Italian Health Ministry, Health Planning Dept): Italian Ministry of Health, Dept of Health Planning: Donata Bellentani, Simona Carbone (technical coordinator), Carla Ceccolini, Angela De Feo, Cristina Giordani, Rosanna Mariniello, Modesta Visca; Dept of health prevention: Natalia Magliocchetti, Giovanna Romano; External Expert: Antonio Lora, Paola Pisanti, Rinaldo Zanini. Polytechnic University of Marche: Flavia Carle (scientific coordinator), Marica Iommi, Edlira Skrami. University of Milano-Bicocca, Laboratory of Healthcare Research and Pharmacoepidemiology: Anna Cantarutti, Giovanni Corrao, Matteo Monzio Compagnoni, Pietro Pugni, Federico Rea. Department of Epidemiology Lazio Region: Marina Davoli, Mirko Di Martino, Adele Lallo. Aosta Valley Region: Patrizia Vittori, Giuliana Vuillermin. Campania Region: Alfonso Bernardo, Anna Frusciante. Emilia-Romagna Region: Laura Belotti, Rossana De Palma. Friuli-Venezia Giulia Region: Andrea Di Lenarda, Marisa Prezza. Lazio Region: Danilo Fusco, Chiara Marinacci. Lombardy Region: Francesco Bortolan, Olivia Leoni. Marche Region: Liana Spazzafumo, Simone Pizzi. Molise Region: Lolita Gallo, Riccardo Tamburro. Puglia Region: Ettore Attolini, Vito Lepore. Sicily Region: Salvatore Scondotto, Giovanni De Luca. Tuscany Region: Paolo Francesconi, Carla Rizzuti. Veneto Region: Francesco Avossa, Silvia Vigna. Research and Health Foundation (Fondazione ReS-Ricerca e Salute-): Letizia Dondi, Nello Martini, Antonella Pedrini, Carlo Piccinni. National Agency for Regional Health Services: Mimma Cosentino, Maria Grazia Marvulli. ANMCO (National Association of Hospital Cardiologists) Study Center: Aldo Maggioni.

## Author contributions

GC, AL, and MMC contributed to the initial study idea and protocol, interpretation of the results, and drafting of the manuscript. MMC contributed to the preparation of the dataset for the analysis and performed all the data analysis. SS, AS, and AG contributed to abstracting the data and authorizing their use and to the interpretation of the results. AB, BD’A, TDF, LF, VT, FC, SC, DC, and AL contributed to the interpretation of pharmacological and clinical prospective results and reviewed the manuscript. GC and MMC were the guarantor of this work and, as such, had full access to all the data in the study and take responsibility for the integrity of the data and the accuracy of the data analysis. All the authors contributed to the critical revision of the manuscript.

## Footnotes

1
https://www.whocc.no/atc_ddd_index/

## References

[1] Jannati A Sadeghi V Imani A Saadati M. Effective coverage as a new approach to health system performance assessment: a scoping review. *BMC Health Serv Res.* (2018) 18:886. 10.1186/s12913-018-3692-7 30470214 PMC6251131

[2] Colston J. *The Use of Effective Coverage in the Evaluation of Maternal and Child Health Programs: a Technical Note for the IDB’s Social Protection and Health Division [Internet].* Washington, DC: Inter-American Development Bank (2011).

[3] Jordans MJD Chisholm D Semrau M Upadhaya N Abdulmalik J Ahuja S Indicators for routine monitoring of effective mental healthcare coverage in low- and middle-income settings: a Delphi study. *Health Policy Plan.* (2016) 31:1100–6. 10.1093/heapol/czw040 27107294

[4] De Silva MJ Lee L Fuhr DC Rathod S Chisholm D Schellenberg J Estimating the coverage of mental health programmes: a systematic review. *Int J Epidemiol.* (2014) 43:341–53. 24760874 10.1093/ije/dyt191 PMC3997372

[5] World Health Organization [WHO]. *WHO Mental Health Action Plan 2013 – 2020.* Geneva: World Health Organization (2012).

[6] Ferrannini L Ghio L Gibertoni D Lora A Tibaldi G Neri G Thirty-five years of community psychiatry in Italy. *J Nerv Ment Dis.* (2014) 202:432–9. 10.1097/NMD.0000000000000141 24821278

[7] Volpe U Fiorillo A Luciano M Del Vecchio V Palumbo C Calò S Pathways to mental health care in Italy: results from a multicenter study. *Int J Soc Psychiatry.* (2014) 60:508–13. 24051155 10.1177/0020764013501648

[8] Tanahashi T. Health service coverage and its evaluation. *Bull World Health Organ.* (1978) 56:295–303. 96953 PMC2395571

[9] Evans D. *Health Systems Performance Assessment: Debates, Methods and Empiricism.* Geneva: World Health Organization (2003).

[10] Ng M Fullman N Dieleman JL Flaxman AD Murray CJL Lim SS. Effective coverage: a metric for monitoring Universal Health Coverage. *PLoS Med.* (2014) 11:e1001730. 10.1371/journal.pmed.1001730 25243780 PMC4171091

[11] Charlson FJ Ferrari AJ Santomauro DF Diminic S Stockings E Scott JG Global epidemiology and burden of schizophrenia: findings from the global burden of disease study 2016. *Schizophr Bull.* (2018) 44: 1195–203. 29762765 10.1093/schbul/sby058 PMC6192504

[12] Pilon D Patel C Lafeuille MH Zhdanava M Lin D Côté-Sergent A Prevalence, incidence and economic burden of schizophrenia among Medicaid beneficiaries. *Curr Med Res Opin.* (2021) 37:1811–9. 34281472 10.1080/03007995.2021.1954894

[13] Rehm J Shield KD. Global burden of disease and the impact of mental and addictive disorders. *Curr Psychiatry Rep.* (2019) 21:10. 10.1007/s11920-019-0997-0 30729322

[14] Charlson F van Ommeren M Flaxman A Cornett J Whiteford H Saxena S. New WHO prevalence estimates of mental disorders in conflict settings: a systematic review and meta-analysis. *Lancet.* (2019) 394:240–8. 31200992 10.1016/S0140-6736(19)30934-1 PMC6657025

[15] Corrao G Barbato A D’Avanzo B Di Fiandra T Ferrara L Gaddini A Does the mental health system provide effective coverage to people with schizophrenic disorder? A self-controlled case series study in Italy. *Soc Psychiatry Psychiatr Epidemiol.* (2021) 57:519–29. 10.1007/s00127-021-02114-9 34132836 PMC8934324

[16] Ciardullo S Rea F Savaré L Morabito G Perseghin G Corrao G. Prolonged use of proton pump inhibitors and risk of type 2 diabetes: results from a large population-based nested case-control study. *J Clin Endocrinol Metab.* (2022) 107:e2671–9. 10.1210/clinem/dgac231 35428888 PMC9202701

[17] Corrao G Soranna D Merlino L Monzani E Viganò C Lora A. Do patterns of mental healthcare predict treatment failure in young people with schizophrenia? Evidence from an Italian population-based cohort study. *BMJ Open.* (2015) 5:e007140. 10.1136/bmjopen-2014-007140 26041489 PMC4458586

[18] Lora A Monzani E Ibrahim B Soranna D Corrao G. Routine quality care assessment of schizophrenic disorders using information systems. *Int J Qual Health Care.* (2016) 28:728–33. 27578632 10.1093/intqhc/mzw096

[19] Lora A Monzio Compagnoni M Allevi L Barbato A Carle F D’avanzo B The quality of mental health care delivered to patients with schizophrenia and related disorders in the Italian mental health system. The QUADIM project: a multi-regional Italian investigation based on healthcare utilisation databases. *Epidemiol Psychiatr Sci.* (2022) 31:e15. 10.1017/S2045796022000014 35156603 PMC8851066

[20] Orrico-Sánchez A López-Lacort M Muñoz-Quiles C Sanfélix-Gimeno G Díez-Domingo J. Epidemiology of schizophrenia and its management over 8-years period using real-world data in Spain. *BMC Psychiatry.* (2020) 20:149. 10.1186/s12888-020-02538-8 32248839 PMC7132863

[21] Burns T. Hospitalisation as an outcome measure in schizophrenia. *Br J Psychiatry Suppl.* (2007) 50:s37–41. 18019042 10.1192/bjp.191.50.s37

[22] Olivares JM Sermon J Hemels M Schreiner A. Definitions and drivers of relapse in patients with schizophrenia: a systematic literature review. *Ann Gen Psychiatry.* (2013) 12:32. 10.1186/1744-859X-12-32 24148707 PMC4015712

[23] Corrao G Rea F Carle F Di Martino M De Palma R Francesconi P Measuring multimorbidity inequality across Italy through the multisource comorbidity score: a nationwide study. *Eur J Public Health.* (2020) 30:916–21. 10.1093/eurpub/ckaa063 32433750

[24] Corrao G Rea F Di Martino M De Palma R Scondotto S Fusco D Developing and validating a novel multisource comorbidity score from administrative data: a large population-based cohort study from Italy. *BMJ Open.* (2017) 7:e019503. 10.1136/bmjopen-2017-019503 29282274 PMC5770918

[25] Farrington CP. Relative incidence estimation from case series for vaccine safety evaluation. *Biometrics.* (1995) 51:228–35. 7766778

[26] Hallas J Pottegård A. Use of self-controlled designs in pharmacoepidemiology. *J Intern Med.* (2014) 275:581–9. 24635348 10.1111/joim.12186

[27] Petersen I Douglas I Whitaker H. Self controlled case series methods: an alternative to standard epidemiological study designs. *BMJ.* (2016) 354:i4515. 10.1136/bmj.i4515 27618829

[28] Whitaker HJ Hocine MN Farrington CP. The methodology of self-controlled case series studies. *Stat Methods Med Res.* (2009) 18:7–26. 18562396 10.1177/0962280208092342

[29] Whitaker HJ Farrington CP Spiessens B Musonda P. Tutorial in biostatistics: the self-controlled case series method. *Stat Med.* (2006) 25:1768–97. 10.1002/sim.2302 16220518

[30] Waljee AK Rogers MAM Lin P Singal AG Stein JD Marks RM Short term use of oral corticosteroids and related harms among adults in the United States: population based cohort study. *BMJ.* (2017) 357:j1415. 10.1136/bmj.j1415 28404617 PMC6284230

[31] Arfè A Corrao G. The lag-time approach improved drug-outcome association estimates in presence of protopathic bias. *J Clin Epidemiol.* (2016) 78:101–7. 10.1016/j.jclinepi.2016.03.003 26976053

[32] Horwitz RI Feinstein AR. The problem of ‘protopathic bias’ in case-control studies. *Am J Med.* (1980) 68:255–8. 10.1016/0002-9343(80)90363-0 7355896

[33] Rea F Corrao G Merlino L Mancia G. Early cardiovascular protection by initial two-drug fixed-dose combination treatment vs. monotherapy in hypertension. *Eur Heart J.* (2018) 39:3654–61. 10.1093/eurheartj/ehy420 30060044

[34] Sansone RA Sansone LA. Antidepressant adherence: are patients taking their medications? *Innov Clin Neurosci.* (2012) 9:41–6. 22808448 PMC3398686

[35] Rush AJ Thase ME. Improving depression outcome by patient-centered medical management. *Am J Psychiatry.* (2018) 175:1187–98. 30220219 10.1176/appi.ajp.2018.18040398

[36] Lacro JP Dunn LB Dolder CR Leckband SG Jeste DV. Prevalence of and risk factors for medication nonadherence in patients with schizophrenia: a comprehensive review of recent literature. *J Clin Psychiatry.* (2002) 63: 892–909. 12416599 10.4088/jcp.v63n1007

[37] Misdrahi D Tessier A Swendsen J Berna F Brunel L Capdevielle D Determination of adherence profiles in schizophrenia using self-reported adherence: results from the FACE-SZ dataset. *J Clin Psychiatry.* (2016) 77:e1130–6. 10.4088/JCP.15m10115 27780318

[38] Ceraso A Lin JJ Schneider-Thoma J Siafis S Tardy M Komossa K Maintenance treatment with antipsychotic drugs for schizophrenia. *Cochrane Database Syst Rev.* (2020) 8:CD008016. 10.1002/14651858.CD008016.pub3 PMC9702459 32840872

[39] Harris EC Barraclough B. Suicide as an outcome for mental disorders. A meta-analysis. *Br J Psychiatry J Ment Sci.* (1997) 170:205–28. 10.1192/bjp.170.3.205 9229027

[40] Lingam R Scott J. Treatment non-adherence in affective disorders. *Acta Psychiatr Scand.* (2002) 105:164–72. 11939969 10.1034/j.1600-0447.2002.1r084.x

[41] Joyce AS Wild TC Adair CE McDougall GM Gordon A Costigan N Continuity of care in mental health services: toward clarifying the construct. *Can J Psychiatry Rev Can Psychiatr.* (2004) 49:539–50. 10.1177/070674370404900805 15453103

[42] Lu EY Cheng ASK Tsang HWH Chen J Leung S Yip A Psychoeducation, motivational interviewing, cognitive remediation training, and/or social skills training in combination for psychosocial functioning of patients with schizophrenia spectrum disorders: a systematic review and meta-analysis of randomized controlled trials. *Front Psychiatry.* (2022) 13:899840. 10.3389/fpsyt.2022.899840 36245879 PMC9561245

[43] Davidson JRT. Major depressive disorder treatment guidelines in America and Europe. *J Clin Psychiatry.* (2010) 71(Suppl E1):e04. 10.4088/JCP.9058se1c.04gry 20371031

[44] Hautala-Jylhä PL Nikkonen M Jylhä J. Continuity of care in psychiatric post-ward outpatient services–conceptions of patients and personnel concerning factors contributing to the continuity of care. *J Psychiatr Ment Health Nurs.* (2005) 12:38–50. 10.1111/j.1365-2850.2004.00790.x 15720496

[45] Hengartner MP. Methodological flaws, conflicts of interest, and scientific fallacies: implications for the evaluation of antidepressants’ efficacy and harm. *Front Psychiatry.* (2017) 8:275. 10.3389/fpsyt.2017.00275 29270136 PMC5725408

[46] National Collaborating Centre for Mental Health [UK]. *Borderline Personality Disorder: Treatment and Management [Internet].* Leicester: British Psychological Society (2009). 21796831

[47] Timäus C Meiser M Bandelow B Engel KR Paschke AM Wiltfang J Pharmacotherapy of borderline personality disorder: what has changed over two decades? A retrospective evaluation of clinical practice. *BMC Psychiatry.* (2019) 19:393. 10.1186/s12888-019-2377-z 31830934 PMC6909459

[48] Hasan A Falkai P Lehmann I Gaebel W. Schizophrenia. *Dtsch Arzteblatt Int.* (2020) 117:412–9. 10.3238/arztebl.2020.0412 PMC7477695 32865492

[49] National Health and Medical Research Council. *Clinical Practice Guideline for the Management of Borderline Personality Disorder [Internet].* (2012). Available online at: https://www.nhmrc.gov.au/about-us/publications/clinical-practice-guideline-borderline-personality-disorder#block-views-block-file-attachments-content-block-1 (accessed February 25, 2021).

[50] Fountoulakis KN Yatham L Grunze H Vieta E Young A Blier P The International College of Neuro-Psychopharmacology (CINP) Treatment Guidelines for Bipolar Disorder in Adults (CINP-BD-2017), Part 2: review, grading of the evidence, and a precise algorithm. *Int J Neuropsychopharmacol.* (2017) 20:121–79. 10.1093/ijnp/pyw100 27816941 PMC5409012

[51] Jones C Hacker D Xia J Meaden A Irving CB Zhao S Cognitive behavioural therapy plus standard care versus standard care for people with schizophrenia. *Cochrane Database Syst Rev.* (2018) 12:CD007964. 10.1002/14651858.CD007964.pub2 PMC6517137 30572373

[52] Barbato A D’Avanzo B Parabiaghi A. Couple therapy for depression. *Cochrane Database Syst Rev.* (2018) 6:CD004188. 10.1002/14651858.CD004188.pub3 PMC6513419 29882960

[53] Chilvers C Dewey M Fielding K Gretton V Miller P Palmer B Antidepressant drugs and generic counselling for treatment of major depression in primary care: randomised trial with patient preference arms. *BMJ.* (2001) 322:772–5. 11282864 10.1136/bmj.322.7289.772 PMC30555

[54] Cuijpers P van Straten A van Oppen P Andersson G. Are psychological and pharmacologic interventions equally effective in the treatment of adult depressive disorders? A meta-analysis of comparative studies. *J Clin Psychiatry.* (2008) 69:1675–85. 18945396 10.4088/jcp.v69n1102

[55] Lora A Lesage A Pathare S Levav I. Information for mental health systems: an instrument for policy-making and system service quality. *Epidemiol Psychiatr Sci.* (2017) 26:383–94. 27780495 10.1017/S2045796016000743 PMC6998623

