# Supplementary material for: From contact coverage to effective coverage of community care for patients with severe mental disorders: A real-world investigation from Italy
Source: Front Psychiatry. 2022 Nov 29;13:1014193. doi: 10.3389/fpsyt.2022.1014193 (PMC9744794; doi:10.3389/fpsyt.2022.1014193)
Supplement: Supplementary file 1 [file Data_Sheet_1.docx]

# **From contact coverage to effective coverage of community care for patients with severe mental disorders:** [**a real-world investigation from Italy**](https://pubmed.ncbi.nlm.nih.gov/23585064/)

**Running title:** Gap between contact and effective coverage of mental healthcare

Giovanni Corrao**1,2**, Matteo Monzio Compagnoni**1,2,***, Angelo Barbato**3**, Barbara D’Avanzo**3**, Teresa Di Fiandra**4**, Lucia Ferrara**5**, Andrea Gaddini**6**, Alessio Saponaro**7**, Salvatore Scondotto**1,8**, Valeria D. Tozzi**5**, Flavia Carle**1,9**, Simona Carbone**10**, Daniel H. Chisholm**11** and Antonio Lora**1,12** on behalf of the “QUADIM project” and “Monitoring and assessing diagnostic-therapeutic paths (MAP)” working groups of the Italian Ministry of Health

**1** National Centre for Healthcare Research and Pharmacoepidemiology, University of Milano-Bicocca, Milan, Italy

**2** Unit of Biostatistics, Epidemiology and Public Health, Department of Statistics and Quantitative Methods, University of Milano-Bicocca, Milan, Italy

**3** Department of Health Policy, Istituto di Ricerche Farmacologiche Mario Negri IRCCS, Milano, Italy

**4** Previously General Directorate for Health Prevention, Italian Health Ministry, Rome, Italy

**5** Centre of Research on Health and Social Care Management, SDA Bocconi School of Management (Bocconi University), Milan, Italy

**6** Agency for Public Health, Lazio Region, Rome, Italy

**7** General Directorate of Health and Social Policies, Emilia-Romagna Region, Bologna, Italy

**8** Department of Health Services and Epidemiological Observatory, Regional Health Authority, Sicily Region, Palermo, Italy

**9** Center of Epidemiology and Biostatistics, Polytechnic University of Marche, Ancona, Italy

**10** Department of Health Planning, Italian Health Ministry, Rome, Italy

**11** Department of Mental Health and Substance Abuse World Health Organization Geneva, Switzerland

**12** Department of Mental Health and Addiction Services, ASST Lecco, Lecco, Italy

**SUPPLEMENTARY MATERIAL**

**Address for correspondence**: Dr. Matteo Monzio Compagnoni, Division of Biostatistics, Epidemiology and Public Health, Department of Statistics and Quantitative Methods, University of Milano-Bicocca, Street Bicocca degli Arcimboldi, 8, Building U7, 20126 Milan, Italy.
E-mail: [matteo.monziocompagnoni@unimib.it](mailto:matteo.monziocompagnoni@unimib.it)

**Supplementary Table S1.** Diagnostic and therapeutic (ICD-9-CM, ICD-10, and ATC) codes used in the current study for drawing records and fields from Healthcare Utilization databases

| **DEPRESSION** | |
| --- | --- |
|  | **ICD-10 codes**  **(Lombardy)** |
| Depressive episode | F32.* |
| Recurrent depressive disorder | F33.* |
| Dysthymia | F34.1 |
| Other persistent mood [affective] disorders | F34.8 |
| Persistent mood [affective] disorder, unspecified | F34.9 |
| Other recurrent mood [affective] disorders | F38.1 |
| Other specified mood [affective] disorders | F38.8 |
| Unspecified mood [affective] disorder | F39.* |
| Post-traumatic stress disorder | F43.1 |
| Adjustment disorders | F43.2 |
|  | **ICD-9-CM codes**  **(Emilia-Romagna, Lazio and Palermo)** |
| Major depressive disorder, single episode | 296.2 |
| Major depressive disorder, recurrent episode | 296.3 |
| Atypical depressive disorder | 296.82 |
| Unspecified episodic mood disorder | 296.90 |
| Depressive type psychosis | 298.0 |
| Dysthymic disorder | 300.4 |
| Adjustment disorder with depressed mood | 309.0 |
| Prolonged depressive reaction | 309.1 |
| Depressive disorder | 311.* |
| **Drugs** | **ATC codes** |
| Antidepressants | N06A |
| **SCHIZOPHRENIA** | |
|  | **ICD-10 codes**  **(Lombardy)** |
| Schizophrenia | F20.* |
| Schizotypal disorder | F21.* |
| Delusional disorders | F22.* |
| Brief psychotic disorder | F23.* |
| Shared psychotic disorder | F24.* |
| Schizoaffective disorders | F25.* |
| Other psychotic disorder not due to a substance or known physiological condition | F28.* |
| Unspecified psychosis not due to a substance or known physiological condition | F29.* |
|  | **ICD-9-CM codes**  **(Emilia-Romagna, Lazio and Palermo)** |
| Schizophrenic disorders | 295.* |
| Delusional disorders | 297.* |
| Other nonorganic psychoses | 298.2, 298.3, 298.8, 298.9 |
| Psychogenic paranoid psychosis | 298.4 |
| **Drugs** | **ATC codes** |
| Antipsychotic agents | N05A (excluded N05AN) |
| **BIPOLAR DISORDER** | |
|  | **ICD-10 codes**  **(Lombardy)** |
| Manic episode | F30.* |
| Bipolar affective disorder | F31.* |
| Cyclothymia | F34.0 |
| Other single mood [affective] disorders | F38.0 |
|  | **ICD-9-CM codes**  **(Emilia-Romagna, Lazio and Palermo)** |
| Bipolar I disorder, single manic episode | 296.0 |
| Manic disorder, recurrent episode | 296.1 |
| Bipolar I disorder, most recent episode (or current) manic | 296.4 |
| Bipolar I disorder, most recent episode (or current) depressed | 296.5 |
| Bipolar I disorder, most recent episode (or current) mixed | 296.6 |
| Bipolar I disorder, most recent episode (or current) unspecified | 296.7 |
| Bipolar disorder, unspecified | 296.80 |
| Atypical manic disorder | 296.81 |
| Other | 296.89 |
| Other specified episodic mood disorder | 296.99 |
| Excitative type psychosis | 298.1 |
| **Drugs** | **ATC codes** |
| Lithium | N05AN |
| Lamotrigine | N03AX09 |
| Valproic acid, Carbamazepine | N03AG01, N03AF01 |
| Second generation antipsychotics (SGAs) |  |
| Olanzapine | N05AH03 |
| Quetiapine | N05AH04 |
| Aripiprazole | N05AX12 |
| **PERSONALITY DISORDER** | |
|  | **ICD-10 codes**  **(Lombardy)** |
| Specific personality disorders | F60.* |
| Mixed and other personality disorders | F61.* |
|  | **ICD-9-CM codes**  **(Emilia-Romagna, Lazio and Palermo)** |
| Personality disorders | 301.* |
| **Drugs** | **ATC codes** |
| Lithium | N05AN |
| Lamotrigine | N03AX09 |
| Valproic acid, Carbamazepine | N03AG01, N03AF01 |
| Second generation antipsychotics (SGAs) |  |
| Olanzapine | N05AH03 |
| Quetiapine | N05AH04 |
| Aripiprazole | N05AX12 |

**Supplementary Table S2.** Service interventions and activities classified in the Italian Mental Health Information System

| **Psychosocial interventions** | **Italian Mental Health Information system codes** |
| --- | --- |
|  |  |
| Individual living skills training | 16 |
| Group living skills training | 17 |
| Individual socialization | 18 |
| Group socialization | 19 |
| Single family psychoeducation | 13 |
| Multifamily group psychoeducation | 14 |
| Individual bodywork (i.e., expressive, practical manual and motor intervention), leisure activities | 21 |
| Group bodywork (i.e., expressive, practical manual and motor intervention), leisure activities | 22 |
| Work training | 23 |
| Assistance with financial and welfare procedures | 25 |
| **Psychotherapy** |  |
| Psychological interview | 02 |
| Individual psychotherapy | 07 |
| Couple psychotherapy | 08 |
| Family psychotherapy | 09 |
| Group psychotherapy | 10 |
| **Generic care** |  |
| Psychiatric visit | 01 |
| Meeting with a professional | 03 |
| Support | 24 |
| Meeting with relatives | 12 |
| Consultation | 04 |
| Medico-legal assessment | 05 |

**Figure S1**. Graphical representation of self-controlled case-referent series

**Footnote**. At first sight, a simple cohort design should be adopted for investigating the association between time-varying exposure to MHC and outcome onset. However, since relevant data such severity of mental health disorder, comorbidities and lifestyle factors (among others), are not measured in a study based on HCU data, a cohort approach likely generates estimates affected by between-person confounding. This happens because more severe patients are expected to receive more likely timely and continuative care, but also more often to experience the outcome. In this way, a paradox positive association between care and outcome is likely generated.

The rationale for the self-controlled case series (SCCS) design is as follows. First, because it is a within-patient design, confounders that do not change over time do not affect SCCS estimates. Second, SCCS involves a between periods comparison, that is it aims to compare the outcome incidence rates observed during person-time spent by each included patient covered and uncovered by MHC. By making within-person comparisons of incidence rates between MHC exposed and unexposed time intervals, SCCS implicitly adjust for all time-invariant individual-level risk factors and potential confounders (measured and not measured). Under the null hypothesis (that is, by hypothesizing that MHC does not affect the risk of relapse), there is not any apparent reason why relapse episodes should occur more frequently when a given patient is not covered by MHC rather than during periods of coverage. Under the alternative hypothesis, conversely, we expect that relapses mainly occur when the patient is not covered by MHC, having he/she abandoned (or discontinued) care.

In the portray of **Figure S1**, **Scenario A**, two relapse events occurred during seven units of person-time on MHC coverage (2/7), and one event occurred during thirteen units uncovered by MHC (1/13), being the corresponding unadjusted crude IRR_c_ = 3.71. This is a rather stranger finding since, under a causal perspective, it could be understood that MHC increases the risk of relapse. It is widely known, however, that validity of SCCS estimates relies on several key assumptions.

Correct allocation of person-time according with MHC coverage is a first key assumption of SCCS design. For example, a patient who experience severe relapse is expected to be more closely followed at once after the outcome is experienced than in other periods. In these conditions, even under the null, width of person-time covered by MHC is expected to be artificial increased after relapse occurring, so generating biased estimate towards positive exposure → outcome association. We attempted of mitigating this effect by excluding from the observational time-window a period of predefined width after each discharge occurred. In the portray of **Figure S1, Scenario B**, one unit of person-time was removed after hospital discharge occurs, so dragging incidence rates to 2/6 and 1/11 and the corresponding IRR_c_ = 3.67.

Another example of exposure misclassification concerns the so-called protopathic bias. As worsening symptoms (i.e., the true but unobservable outcome) likely requires more intensive MHC and increases the risk of the detected outcome (hospital discharge), person-time covered by MHC is expected to be artificial increased before relapse occurring, so generating biased estimates towards positive exposure → outcome association. We attempted of mitigating this effect by excluding from the observational time-window a period before outcome occurs, that is the outcome occurrence was artificially anticipated. In the portray of **Figure S1, Scenario C**, one unit of person-time was removed before each hospital admission, so dragging incidence rates to 1/4 and 2/10 and the corresponding IRR_c_ = 1.25.

Other than the correct exposure classification, absence or irrelevance of confounding should be verified. In fact, although SCCS eliminates confounding by between-person characteristics that remain stable over time, in order to attain valid results, differences in the probability of exposure and outcome that change over time must be addressed in the design and analysis of the study. As a motivating example, let consider the time as potential confounder. In our application, time trends in experiencing both exposure and outcome of target population (newly-taken-in-care patients with mental health disorders) may remarkably influence the estimates. In fact, a clear concentration of both MHC and outcomes was observed early after diagnosis, the corresponding rates being gradually decreased afterwards. In these conditions, an artificial positive exposure → outcome association should be observed for the easy reason that exposure and outcome are time-correlated. We tried of accounting for this source of bias by means of an original approach consisting in combining the self-controlled case series as above described with a self-controlled referent series (the corresponding approach being labelled as self-controlled case-referent series (SCCRS) design). This was made by randomly selecting from the same cohort which generated the case series, cohort members who during the observational time-window experienced periods covered, as well as periods uncovered, by exposure to MHC, but did not experience any relapse episode. Case and referent series were individually matched for gender, age at cohort entry (±2 years), and date of mental health disorder diagnosis (±60 days). Referent patients were assumed to experience the outcome when the matched case suffered from it. In addition, referent series cohort was submitted to the same criteria of person-time exclusion as the case series cohort as above discussed. From the self-controlled referent series so built, a referent incident rate ratio (IRR_r_) was calculated. Because referent patients did not experience the outcome, IRR_r_ is expected to be unaffected by the exposure → outcome association, but rather to the portions of IRR_c_ due to time-trend. By dividing IRR_c_ by IRR_r_, an IRR_a_ adjusted for time-trend was obtained. The portray of **Figure S1, Scenario D**, shows referent incidence rates of 2/4 and 1/10 respectively expected during exposure and non-exposure periods, so that an IRR_r_ = 5.00 is expected under the null. The ratio between the incidence rate ratio observed for case series (IRR_c_ = 1.25; **Scenario C**) and that observed for referent series (IRR_r_ = 5.00; **Scenario D**), generates an IRR_a_ = 0.25, that is coverage with MHC is associated with a 75% reduced risk of relapse.

Despite the use of this expedient, however, time varying confounders might yet affect estimates. For example, let address the effect of psychotherapy. Patients who had both periods of coverage and no-coverage with psychotherapy should be included according with the proposed approach. However, exposure to drug therapy could be unbalanced between the compared periods, being medicaments likely more extensively dispensed when psychotherapy is active. In these conditions, drug exposure might confound the effect of psychotherapy. In addition, the effect of other time-varying covariates might be surrogated by exposure to psychotherapy, for example by other unintended care sources. Based on these premises, in the current application IRR_c_ and IRRr were adjusted according with the rates of exposure to (i) MHC other than that of specific interest (i.e., to drug therapy, to other psychosocial interventions, and to other no-psychotherapeutic, no-psychosocial interventions when the effect of psychotherapy was investigated), (ii) hospital admissions, and drug and other any outpatient services unrelated with mental health dispensed to each cohort member during the compared periods.

**Supplementary Table S3.** Self-controlled case-referent series estimates for the effect of antidepressants, antipsychotics, and mood stabilizers on the risk of relapse in patients with diagnosis of personality disorders

| **Pharmacotherapies** | **IRR (95% CI)** |
| --- | --- |
| All together | 1.18 (0.94 to 1.49) |
| Antipsychotics | 1.01 (0.78 to 1.30) |
| Antidepressants | 1.37 (0.63 to 2.94) |
| Mood stabilizers | 1.22 (0.89 to 1.65) |

**Supplementary Table S4.** Sensitivity analysis of self-controlled case-referent series estimates. Box A: a 90-day width time-window prior the relapse onset was removed (rather than of 180 days as in the main analysis). Box B: only the first relapse episode was considered (rather than all the relapses as in the main analysis)

1. **Reducing the width of the removed time-window prior the relapse onset**

|  | Depression | Schizophrenia | Bipolar disorder | Personality disorder |
| --- | --- | --- | --- | --- |
| Drug therapy | 0.97  (0.80 to 1.17) | 0.64  (0.54 to 0.76) | 0.56  (0.42 to 0.74) | 1.18  (0.94 to 1.48) |
| Generic MHC  (no psychosocial interventions) | 1.31  (1.11 to 1.53) | 1.04  (0.89 to 1.22) | 1.18  (0.91 to 1.53) | 1.68  (0.65 to 4.38) |
| Psychosocial interventions (no psychoterapy) | 0.80  (0.60 to 1.06) | 0.90  (0.70 to 1.16) | 0.74  (0.49 to 1.12) | 1.11  (0.84 to 1.46) |
| Psychotherapy sessions | 0.76  (0.56 to 1.03) | 0.79  (0.56 to 1.10) | 0.83  (0.50 to 1.39) | 1.13  (0.84 to 1.53) |

1. **Censoring information at the first episode of relapse**

|  | Depression | Schizophrenia | Bipolar disorder | Personality disorder |
| --- | --- | --- | --- | --- |
| Drug therapy | 1.04  (0.77 to 1.40) | 0.71  (0.52 to 0.97) | 0.77  (0.45 to 1.31) | 1.08  (0.77 to 1.74) |
| Generic MHC  (no psychosocial interventions) | 1.07  (0.82 to 1.39) | 0.92  (0.69 to 1.22) | 0.82  (0.51 to 1.33) | 1.65  (0.93 to 2.93) |
| Psychosocial interventions (no psychoterapy) | 0.66  (0.38 to 1.16) | 0.66  (0.45 to 0.96) | 0.85  (0.43 to 1.70) | 0.61  (0.40 to 0.94) |
| Psychotherapy sessions | 0.58  (0.32 to 1.03) | 0.47  (0.29 to 0.76) | 0.60  (0.24 to 1.49) | 0.54  (0.33 to 0.89) |
