# Supplementary figures and images for: From contact coverage to effective coverage of community care for patients with severe mental disorders: A real-world investigation from Italy
Source: Front Psychiatry. 2022 Nov 29;13:1014193. doi: 10.3389/fpsyt.2022.1014193 (PMC9744794; doi:10.3389/fpsyt.2022.1014193)

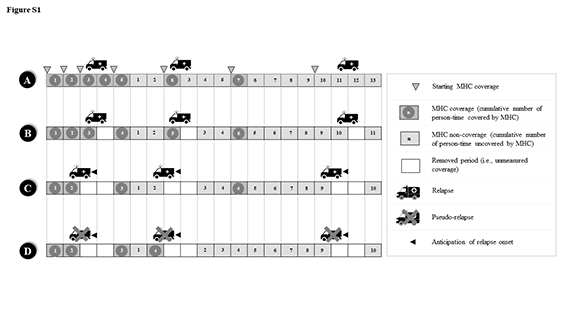

Supplement: Supplementary file 2 [file Image_1.TIF]
